# Case Report: Nonverbal approaches in the treatment of a patient with fibromyalgia with anger rooted in adverse childhood experiences

**DOI:** 10.3389/fpain.2024.1374324

**Published:** 2024-05-16

**Authors:** Yuri Adachi, Masako Hosoi, Tomoe Nishihara, Naoki Hirabayashi, Takako Sawa, Tomoko Matsushita, Keita Tatsushima, Kozo Anno, Mitsunao Tomioka, Nobuyuki Sudo

**Affiliations:** ^1^ The Health Center of Fukuoka Dental College and Fukuoka Nursing College, Fukuoka College of Health Sciences, Fukuoka, Japan; ^2^ Department of Psychosomatic Medicine, Kyushu University Hospital, Fukuoka, Japan; ^3^ Multidisciplinary Pain Center, Kyushu University Hospital, Fukuoka, Japan; ^4^ Department of Psychosomatic Medicine, Graduate School of Medical Sciences, Kyushu University, Fukuoka, Japan; ^5^ Independent Counseling Facility, Kotonoha Child Developmental Counseling Room, Ebetsu, Japan; ^6^ Center for Health Sciences and Counseling, Kyushu University, Fukuoka, Japan; ^7^ Department of Endocrinology and Metabolism, Toranomon Hospital, Tokyo, Japan; ^8^ Health Support and Counseling Center, Reiwa Health Sciences University, Fukuoka, Japan

**Keywords:** fibromyalgia, ACEs, therapeutic relationship, anger, nonverbal approach, drawing therapy

## Abstract

**Introduction:**

In psychotherapy, it is important to establish and deepen a therapeutic trusting relationship, but patients who have experienced extreme adversity in childhood tend to have difficulty in building such a relationship. This paper reports a case of fibromyalgia with adverse childhood experiences (ACEs) in which a nonverbal approach was successful in building a trusting relationship.

**Case and methods:**

The patient is a woman in her late 40s. She had strong anger rooted in ACEs, including neglect by her father, a feeling of unfair parenting by her mother compared to her younger brother, overcontrol of her life by her mother, and sexual abuse by her uncle. She was filled with strong interpersonal distrust and anger, and the experience of an unsuccessful surgery compounded her distrust of medical care. The therapist initially had severe difficulty in verbal interaction with the patient. When conducting “drawing” therapy, she ignored the therapist's comments and completely blacked out the drawing paper. However, the patient-therapist relationship gradually changed, and verbal interaction became possible through the use of nonverbal approaches such as framing her drawing paper and “Towel Baby Holding.”

**Results:**

The therapist was able to understand the patient's emotions through these nonverbal approaches and to communicate with the patient that she understood her feelings. This approach was also successful in the patient’s understanding of her own pathology. The patient became able to honestly express her feelings in words, which eventually enabled her to be introduced to mindfulness therapy, leading to a favorable treatment course.

**Conclusion:**

For patients with ACEs, a nonverbal approach helps build a therapeutic relationship and plays an important role in understanding the patient.

## 1 Introduction

Adverse Childhood Experiences (ACEs) have been shown to be closely related to physical health problems and mental disorders in adulthood and to difficulties in interpersonal relationships and emotional regulation (1). The relation between ACEs and fibromyalgia has recently been reported (2). Fibromyalgia, a refractory disease characterized by chronic intractable pain and psychological suffering, is currently one of the most difficult types of chronic pain to treat. Although mindfulness therapy, a kind of psychotherapy, has been applied worldwide for fibromyalgia, its usefulness is not consistent (3). One of the reasons for this inconsistency may be the difficulty in establishing a therapeutic relationship with a patient with fibromyalgia and ACEs. It has been noted that patients with ACEs are distrustful (1) and prone to interpersonal problems (4). We have also shown that attachment and distrust are common psychological characteristics in the case of intractable chronic pain (5). Recent studies have shown that working with a strong therapeutic alliance is a prerequisite for the treatment of patients with fibromyalgia (6, 7) and that a negative relationship with a therapist influences the worsening of central sensitivity syndrome symptoms, one manifestation of fibromyalgia (8).

In psychotherapy, it is important to establish and deepen a therapeutic trusting relationship. Patients with a strong distrust of medical care often come to us with an attitude of “I don't want to talk” but “I want to be cured,” and it is often a challenge to lead them to soften their refusal and conflicting attitudes.

Herein, we report a case in which we had difficulty establishing but were able to build a trusting relationship between a patient and her therapist. This was achieved through various approaches, including art therapy with drawings, a nonverbal approach that encourages patients to express themselves (9). Treating a patient with such strong distrust of medical care is difficult because of the difficulty of verbal interaction, and drawing was the only method that could be continued long-term in this case. We discuss how this nonverbal approach worked to build the patient-therapist relationship.

## 2 Background

### 2.1 Patient

Akiko (name changed to provide anonymity), a woman in her late 40s at first visit.

Chief complaint: Pain in both lower limbs, back of the head, shoulders, neck, and collarbone.

### 2.2 History of the current illness

The history of present illness is described in the Supplementary Table S1. At Rehabilitation Hospital A, milnacipran hydrochloride, clotiazepam, sodium valproate, Touki peony, a preparation containing vaccinia virus-inoculated rabbit inflammatory skin extract, diclofenac sodium, and mecobalamin were prescribed, but the medication was discontinued because the symptoms persisted with no response. She was referred to our Department of Psychosomatic Medicine, Kyushu University Hospital for the treatment of fibromyalgia in May of year X.

### 2.3 Family history

Akiko's family consisted of her father, mother, and a younger brother. Her father was often transferred, so she changed schools many times. Her grades were in the upper-middle range. When she was in the sixth grade, her mother became ill, and she was sent to a relative's home in another prefecture. In the first year of junior high school, she was told to refrain from sports due to acetabular dysplasia. After graduating from junior college, she worked at a nursery school. Her parents separated the year she started working there. She worked for about 20 years in a position of responsibility, but she decided to have a hip joint operation and resigned, thinking about her future (marriage, having children) (Supplementary Table S2).

### 2.4 Course of treatment prior to this intervention

In year X, Akiko was hospitalized for the first time in September. Problems such as a strong distrust of medical treatment and conflicts with her parents became apparent. A doctor and a psychologist interviewed her, her symptoms temporarily lessened, and she continued to visit the hospital as an outpatient. In February of year X + 2, she reported being sexually abused by her uncle at a relative's house when she was in elementary school, after which she had hyper arousal and her muscle stiffness and pain became stronger, which led to a diagnosis of post-traumatic stress disorder (PTSD). Akiko was hospitalized for a second time with the above main complaints in April of year X + 2 due to a flare-up of negative feelings toward her mother. She was discharged from the hospital in July of year X + 2 under the care of an outpatient physician and a psychologist.

Psychotherapy was started during the hospitalization in year X, and the first author took over in October of year X + 3. The frequency of sessions was about once every two weeks, with each session lasting 50 min. During the handover, the previous psychologist had recommended drawing because the patient had said that drawing helped her clear her mind and organize her feelings.

## 3 Therapeutic intervention

Based on the “Theory of Intimacy” (10), we divided the treatment flow into four phases.

### 3.1 Phase 1: a period of unawareness of the new therapist [sessions 1 (*X* + 3) −17 (*X* + 4)]

In session 1, a sense of tension immediately arose when Akiko entered the room. After self-introductions, the psychologist (“TH”) spoke and Akiko stared at TH sharply, with her expression becoming more and more severe. Akiko spoke in a burst of words, saying that she had to help her mother because she was not feeling well, but that her mind was not up to it. She left no room for TH to speak. She repeatedly said that she could not trust people and did not like people. When TH tried to respond, she was immediately interrupted. When talking about the abuse by her uncle as a child, Akiko burst into tears and turned away. When TH asked if she would like to draw a picture, without speaking she took a black crayon and completely blacked out the drawing paper (Supplementary Figure S1A). When the crayon was almost completely used, she said that she had wanted to fill in more.

In session 2 and after, there was not much interaction with TH during the sessions. The same sequence of events was reported each time: (1) As a child, her mother disciplined her strictly and she was only accepted if she was good, (2) as a child, the sexual abuse by her uncle, and (3) as an adult, a medical error ruined her life. When she talked about her victimization and abuse, she became emotional and shed tears. After 30–40 min, TH would suggest drawing, but it was difficult for Akiko to switch when she was in an emotional state, and she usually ended the session after 15–25 min. During this time, TH could barely interject, and when she did the patient kept refuting her comments, saying “No, it's not that” or “It's more than that…” although TH had only repeated the patient's words. TH's attempts to summarize or ask questions were denied or ignored. Black was used almost exclusively in the drawings, and the drawings in Supplementary Figures S1B–D went over the edge of the drawing paper.

### 3.2 Phase 2: a period of awareness [sessions 18 (*X* + 4)−26 (*X* + 4)]

In session 18, Akiko reported having recently visiting a local orthopedic doctor due to back pain. When she told him about her medical history, he defended the doctor at Hospital B, and she felt as if she was being criticized. When TH asked if she had been afraid during that visit, she shed tears of affirmation, and TH felt accepted for the first time. When it was suggested that TH put a frame on the drawing paper during the drawing session, she agreed and drew without going beyond the frame, telling TH that she did not mind doing so. Although the content of what she said in her sessions remained the same, her drawing no longer protruded from the drawing paper and colors other than black began to be used (Supplementary Figure S1E). Akiko gradually began to respond to TH’s questions about how she felt, however, there was no change in her one-way talking or exceeding the time frame of the session.

### 3.3 Phase 3: a period of deepening relationship by holding a “towel baby” [sessions 27 (*X* + 4) −29 (*X* + 4)]

In session 27, when the patient began to talk about how she was unreasonably forbidden by her mother to play with others when she was a child, TH felt as if the patient were acting as a young child. Taking a towel from next to the bed in the interview room, TH rolled it up and held it like a baby, saying “This baby has been feeling frustrated, sad, and wanting to play. It has been ignored for a long time.” The patient closely watched TH. As she left the room she suddenly started crying and said “If only someone had done this for me when I was a child, my life might have been different.”

In session 28, Akiko said that when TH stroked the towel, she felt that TH was accepting her. She began to speak effusively without allowing interruption by TH, and the session was extended to allow her to continue talking.

### 3.4 Phase 4: a period of interaction [sessions 30 (*X* + 4)−92 (*X* + 6)]

In session 30, TH told Akiko that she could only take 50 min the next session, and she agreed. In the next session she told TH about a dream she had that took place in her old workplace and in which she had to follow instructions from her supervisor and reluctantly obeyed them. When TH pointed out the similarity between the content of the dream and a previous exchange, she affirmed this and said that she really felt that she did not like it, for the first time expressing her feelings in words. Akiko also told TH honestly that she understood the reasons for not receiving extra time for the sessions, but that she was still struggling and needed more time. TH changed the sessions from every two weeks to every week, and time extensions were no longer necessary.

In session 34, Akiko was able to look back on her feelings, understanding and verbalizing them, saying things such as “I was angry at that time.” She also recognized the differences between the drawings she had made in the early and later sessions (Supplementary Figures S1B,F). Her expression of honest feelings increased, and she was also able to express to TH that she felt pampered, also expressing her feelings of having wanting to be pampered and loved by her parents and wanting to be acknowledged and hugged, feelings that were behind her anger.

In the fall of year *X* + 6, when TH reported that she could not continue the sessions because she was pregnant Akiko expressed joy, but also honestly expressed mixed feelings, ending with a smile and a handshake.

## 4 Discussion

### 4.1 Psychological pathology rooted in ACEs

In the early sessions, the patient was full of anger and clearly distrustful of her therapist and the medical staff. This may have been due to the fact that she had grown up with a nervous and immature mother, a father who did not care about the family, had been treated unequally with her younger brother by her mother, and was sexually abused by her uncle, among other adverse childhood experiences. It has been reported that people with such experiences are more likely to have difficulty with emotional regulation and distrust of others (1, 4). While distrust was high, over-adaptation to gain affection and repression of emotions became the norm, and the patient seemed to exhibit dysphoria, lacking awareness of her own emotional experiences. As a result, although she was experiencing intense feelings of anger, she was not expressing these feelings in an appropriate manner. The combination of trauma from her childhood experiences and medical errors made it difficult to establish a patient-therapist relationship because the patient was prone to anger in interpersonal relationships but was unaware of these feelings.

### 4.2 Difficulties in establishing the patient-therapist relationship

The patient’s previous psychologist was changed due to maternity leave. Although she did not explicitly state it, Akiko acknowledged that she could not hope for a “rebirth (marriage and childbirth)” when she was separated from her psychologist. Feelings of abandonment and envy (for both the previous psychologist and her baby) arose because she had resigned from her job due to her dream of making her own warm family by marriage and having children. Akiko felt that the new psychologist was not reliable, as had been her previous physicians and psychologists. The “drawing in black” (Supplementary Figure S1A) expressed in session1 may have been a projection of anxiety and anger related to that untrustworthiness. However, even after drawing, she did not express her feelings to TH in words. She continued to attend the sessions as promised, but continued to talk about herself incessantly. Her words in session 34, “I realize that I was angry at that time,” indicated that she had lacked awareness of her feelings during the earlier sessions and had not even been aware that she was angry, but that a sense of discomfort was growing in her.

### 4.3 Three walls to her real intention

We feel that there were three levels of barriers (walls) to the development of the patient-therapist relationship. Supplementary Figure S2 shows the three methods used to break the walls.

#### 4.3.1 The first wall: distrust and anger at medical personnel: the method that worked to break it; drawing within a frame

The first turning point was the framing of the drawings. According to Kaido (11), “framing” by the therapist has the following functions: (1) to protect expression, (2) to reveal more of the inner self and hidden desires, (3) to make the subject feel that he/she has to draw, and (4) to make the subject aware of the presence of the therapist.” In the present study, the framing of drawings by TH helped make Akiko, who had been seeking “all-around motherly treatment” and had been “ignored” for a long time, aware of TH’s existence and provided an opportunity for her to feel “protected”, after which she gradually began to verbally interact with TH. When TH asked a question, Akiko thought about it, an interaction that had not been observed before. At the same time, the drawing became less and less out of the frame, and colors other than black or brown became prominent (Supplementary Figure S1E). Emotions other than anger and anxiety began to emerge.

#### 4.3.2 The second wall: anger with her mother and preoccupation with past emotions: the method that worked to break the wall; “towel baby holding”

Even with the changes described above, there was no significant change in what Akiko talked about. The same content was repeated every session, and it was difficult for her to look at herself objectively or to reflect on her own life. At the same time, there were moments when she seemed emotionally like an infant. TH felt that Akiko, who had lamented her mother's unreasonableness in session 27, was like an infant who “wanted to be hugged”, but could not express this feeling in words, so TH rolled up a towel and held it as if “holding a baby”. Iimori describes nonverbal approaches, as a symbolic projection of inner worlds and situations that are unnoticed in verbal communication, which is intuitively conveyed to the other person and helps to deepen understanding and acceptance (12). TH told the patient that her internal world was that of a two or three-year-old and, as an expression of acceptance of her wish to be “accepted as she was,” TH held and stroked the towel, which symbolically stroked her. When the patient left the room, she cried and said, “I really wanted them to do this to me,” which suggested that this nonverbal approach was a means for her to intuitively understand and symbolically comprehend what TH wanted to convey. This suggests that this nonverbal approach was a symbolic means of allowing her to understand what TH wanted to convey.

#### 4.3.3 The third wall: inability to keep within a time frame: the method that worked to break the wall; encouragement of verbalization of feelings

It was difficult for the patient to keep within the set time structure. She understood the framework of 50 min per session and did not show any resistance when she re-signed her contract with TH. However, she usually exceeded the time by 15–25 min, which made TH anxious about the uncertainty of the ending time of each session and also somewhat upset about the uncertainty. After session 27, when she became able to interact with the patient, TH was able to tell her that she could only do 50 min, but the patient was not satisfied with this and resisted.

Through the story of a dream, the patient was able to express her resistance in her own words and to directly tell TH that she wanted her to spend more time with her. The words “I want you to spend more time with me” were an honest expression of her feelings toward the therapist, as were “I want you to understand me more completely,” and were easy for TH to understand. The fact that there was no prolongation of the interview time after the frequency was changed suggests that the patient felt that her thoughts and feelings were accepted.

### 4.4 Patient change

A relationship of trust with the patient was able to be established through the above process. The patient began to actively talk about her feelings, which she had previously been unaware of and unable to verbalize. She also began to express her anger toward her mother and her desire for her mother to pay more attention to her and to pamper her more, the lack of which was behind her anger, and she was able to admit this. These processes are summarized in Supplementary Figure S2. Although she was still not able to express these desires directly to her mother, her improvement was a steppingstone to subsequent treatment through methods such as mindfulness therapy. As the clinical course shows in Supplementary Figure S3, pain experience including negative affectivity (depression and state anxiety) rooted from ACEs contributed to the appearance of her complaints, which is often observed in cases of fibromyalgia. The pain scores measured by short-form McGill Pain Questionnaire (Sensory pain rating index, Affective pain rating index, and Visual Analogue Scale) and the percentiles of The Center for Epidemiologic Studies Depression Scale (CES-D) and State-Trait Anxiety Inventory (STAI) decreased. Akiko initially complained of widespread pain to her doctor, but the complaints stopped after the sessions with the TH: Through the above, Akiko's pain was relieved, and she was able to successfully return to society.

Pasini et al. reported that some of the factors that cause changes in self-efficacy of pain-management, emotional regulation ability, and pain intensity perception occur through an important bond between therapist and patient (7). The reason Akiko's complaints disappeared may be that the flames of anger rooted in her ACEs, which often interfere with mindfulness therapy, were quenched by the bond created by the intensive nonverbal and subsequent verbal counseling. When the problems caused by her strong negative affect; the anger, hate, and jealousy that created her lack of basic trust; had been fully addressed by our psychological interventions, our mindfulness therapy dramatically worked to reduce the pain complaints of this patient.

### 4.5 Significance of the nonverbal approach

Drawing was critical to the treatment of this patient. Communication became possible because of the framing of the relationship through drawing. TH was able to understand through the drawings emotions that could not expressed in words. Conversely, TH was able to convey her presence to Akiko and to gain an understanding of how she felt by appealing visually, rather than verbally, through the frame of the drawing and the “Towel Baby”.

Despite her early strong anger rooted in ACEs and difficulty in establishing a therapeutic relationship, mindfulness was shown to very effective, probably because we took sufficient time for the nonverbal approach to obtain a good therapeutic relation before starting mindfulness therapy.

## 5 Conclusion

Drawing using the framing method and holding a “Towel Baby” were useful in the treatment of this patient with greatly inflated negative emotions rooted in adverse childhood experiences. In some cases, patients who have difficulty with their therapists due to incidents of childhood maltreatment and distrust of medical treatment may not be able to establish an appropriate relationship with the therapist, leading to doctor shopping without completing treatment which is thought to be effective like mindfulness therapy. The use of nonverbal therapies, such as drawing, along with verbal counseling therapies may be effective in the treatment of difficult-to-treat cases, such as this case of fibromyalgia.

## Data Availability

The datasets presented in this article are not readily available because this is a case report and includes only personal data. Requests to access the datasets should be directed to Masako Hosoi, hosoi.masako.642@m.kyushu-u.ac.jp.
