# Supplementary material for: Case Report: Nonverbal approaches in the treatment of a patient with fibromyalgia with anger rooted in adverse childhood experiences
Source: Front Pain Res (Lausanne). 2024 May 16;5:1374324. doi: 10.3389/fpain.2024.1374324 (PMC11137261; doi:10.3389/fpain.2024.1374324)

Table S1
History of Present Illness

Year	History of Present Illness
X-8	Appearance of pain in both knees
X-6	Synovectomy at Hospital A
X-5	Rotational osteotomy at Hospital B
X-4	The doctor in charge at Hospital B confessed that there was a medical error.
X-2	Rotational osteotomy at Hospital C; however, pain in both lower limbs, shoulders, neck, both elbows, the clavicle, and lower back persisted.
X-1	Visited a physician at Hospital C: suspected of having fibromyalgia.
X	Referral visit to the department of psychosomatic medicine in our hospital (D).

X: year of first referral visit to our hospital.
(X-8: 8 years pre-referral to our team.)
Hospital A: A local hospital
Hospital B: A private Japanese university hospital
Hospital C: A private Japanese university hospital far from her residence
Hospital D: A Japanese national university hospital

Table S2
History of Adverse Experiences

History of Adverse Childhood Experiences
Childhood years～<br>Akiko changed schools many times.<br>Neglect by her father<br>Psychological abuse by her mother<br>(Her father was rarely at home. Her mother was nervous, always crying, and overly controlling of her life)
5 YO~ Birth of a younger brother<br>(Her mother was strict with her and lenient with her younger brother)
12YO Onset of the mother's brain disease<br>Sexual abuse by her uncle
13YO Refrained from sports due to acetabular dysplasia
**History of Adverse Adult Experiences**
20YO～Parents separated<br>Began teaching career
30s～Psychological harassment by an orthopaedic doctor and her sister-in-law

Figure S1 Drawings

a.#1 (Fully blacked out drawing)

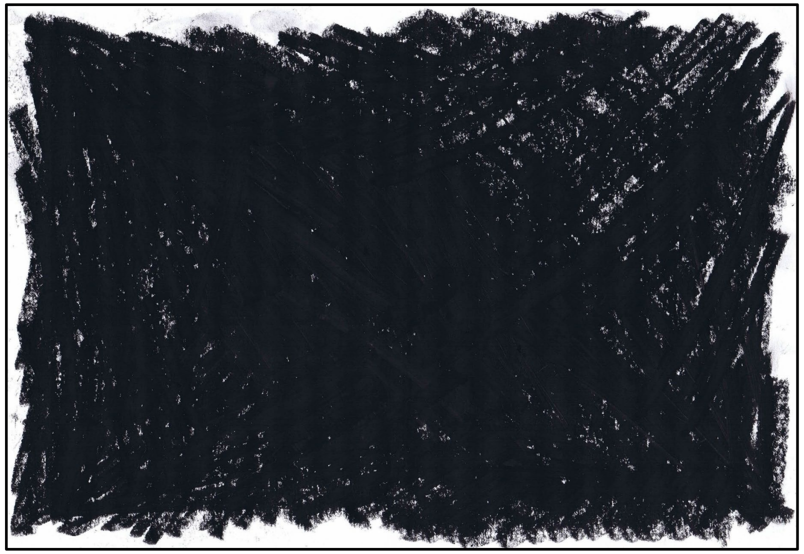

b.#4 Tree

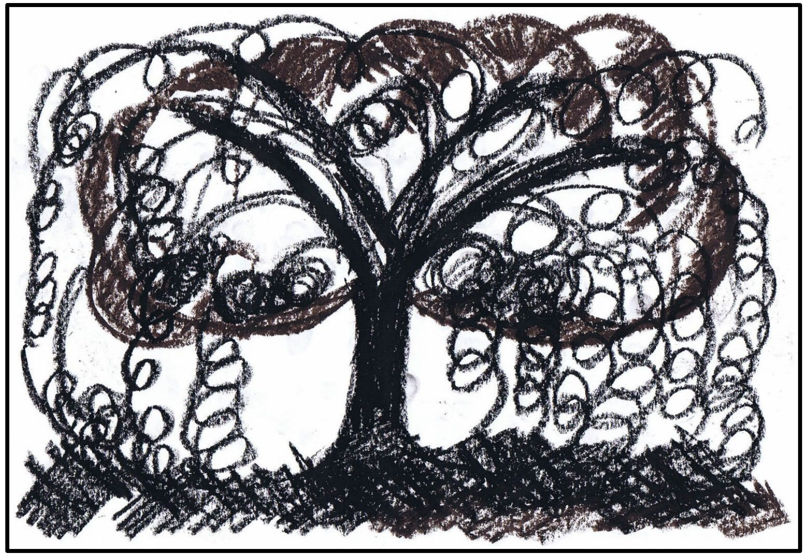

c.#14 Surrounding eyes (off the paper)

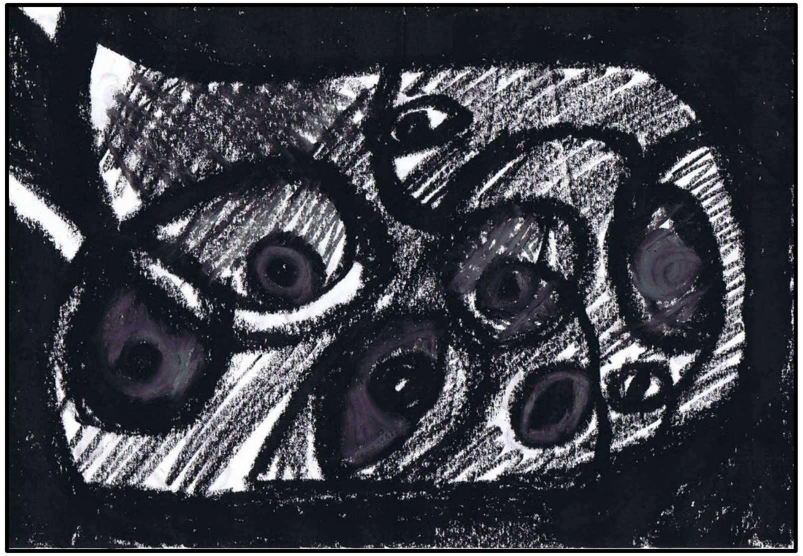

d.#16 People blame me (off the paper)

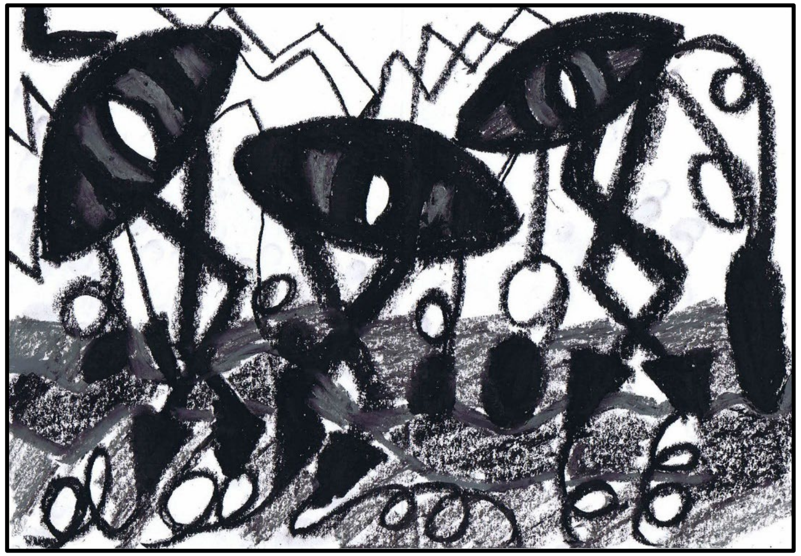

e.#28 Colorful feeling (within a frame)

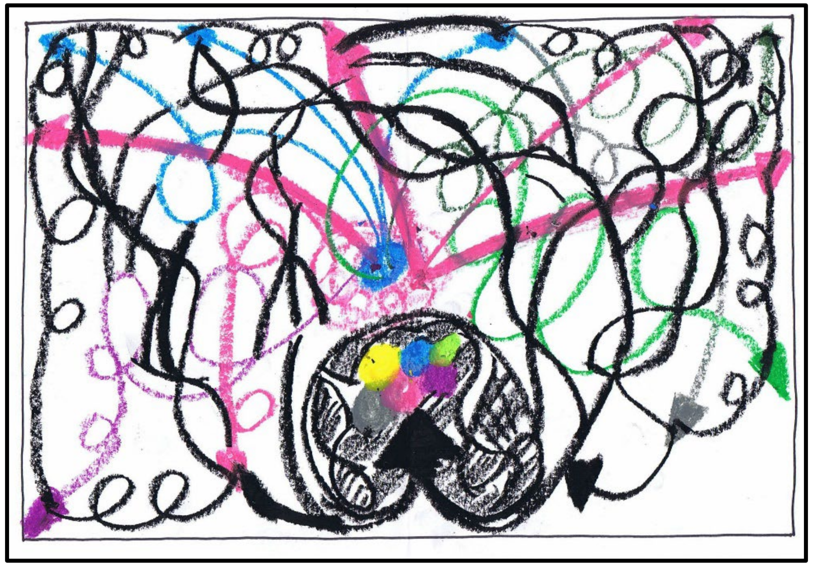

f.#31 Colored Tree (within a frame)

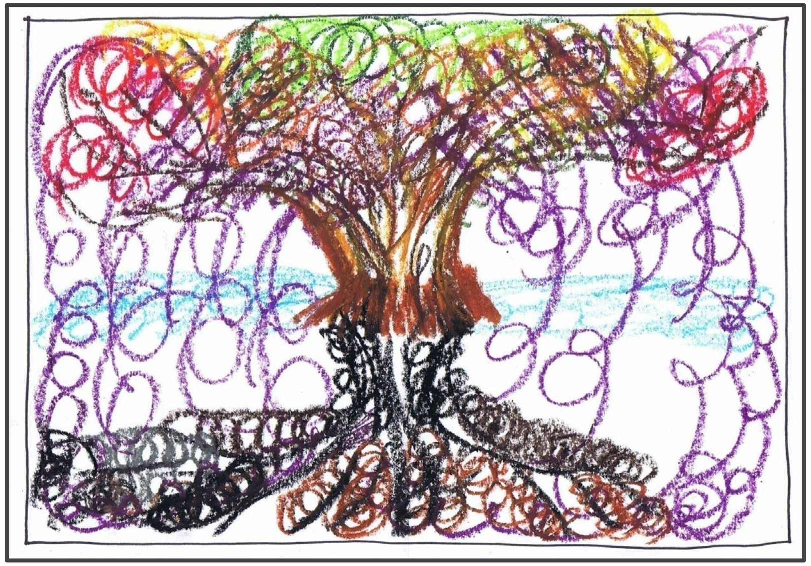

Figure S2. Progression of Treatment

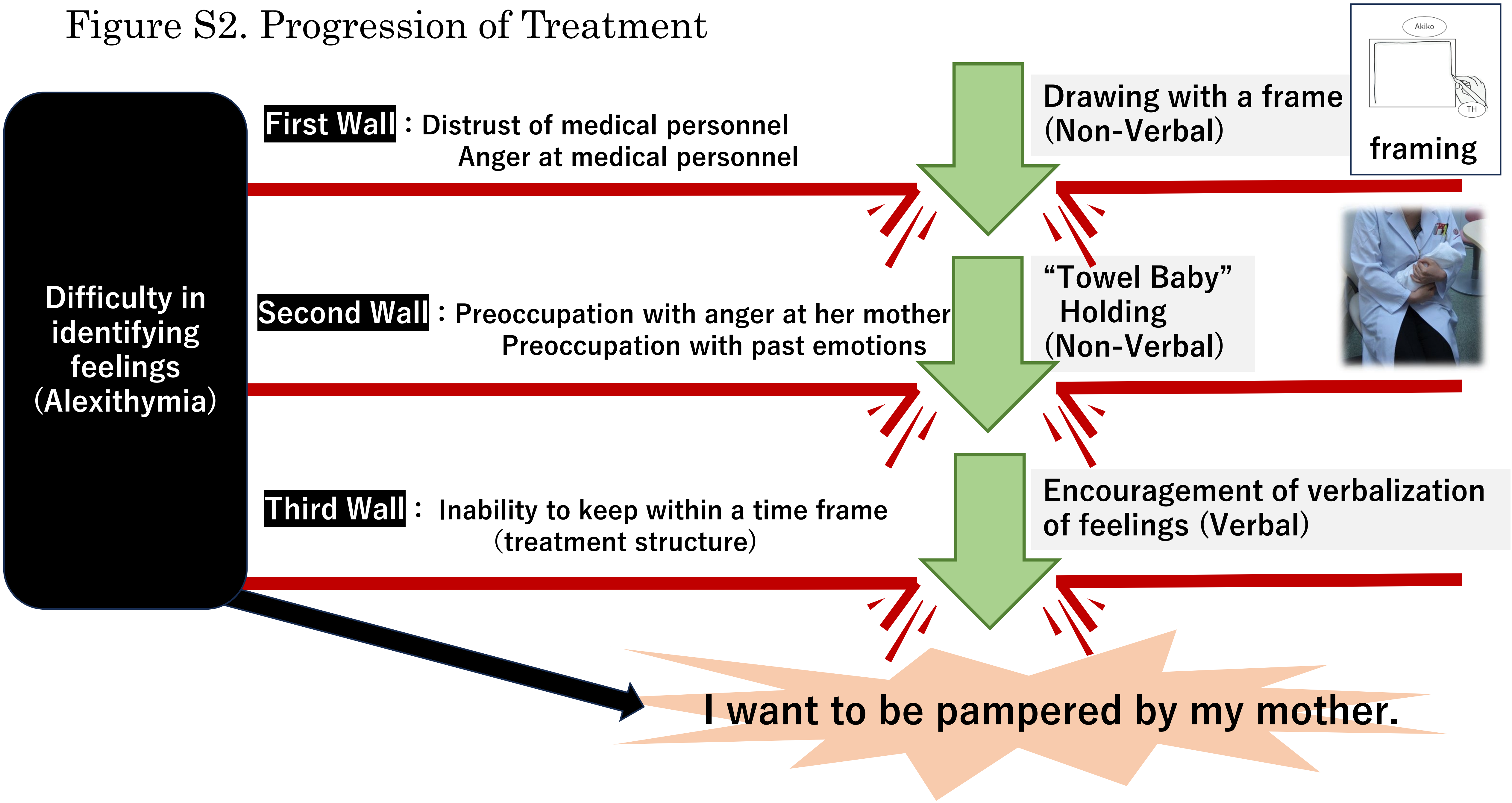

Figure S3. Depression and anxiety percentile and pain scores over time

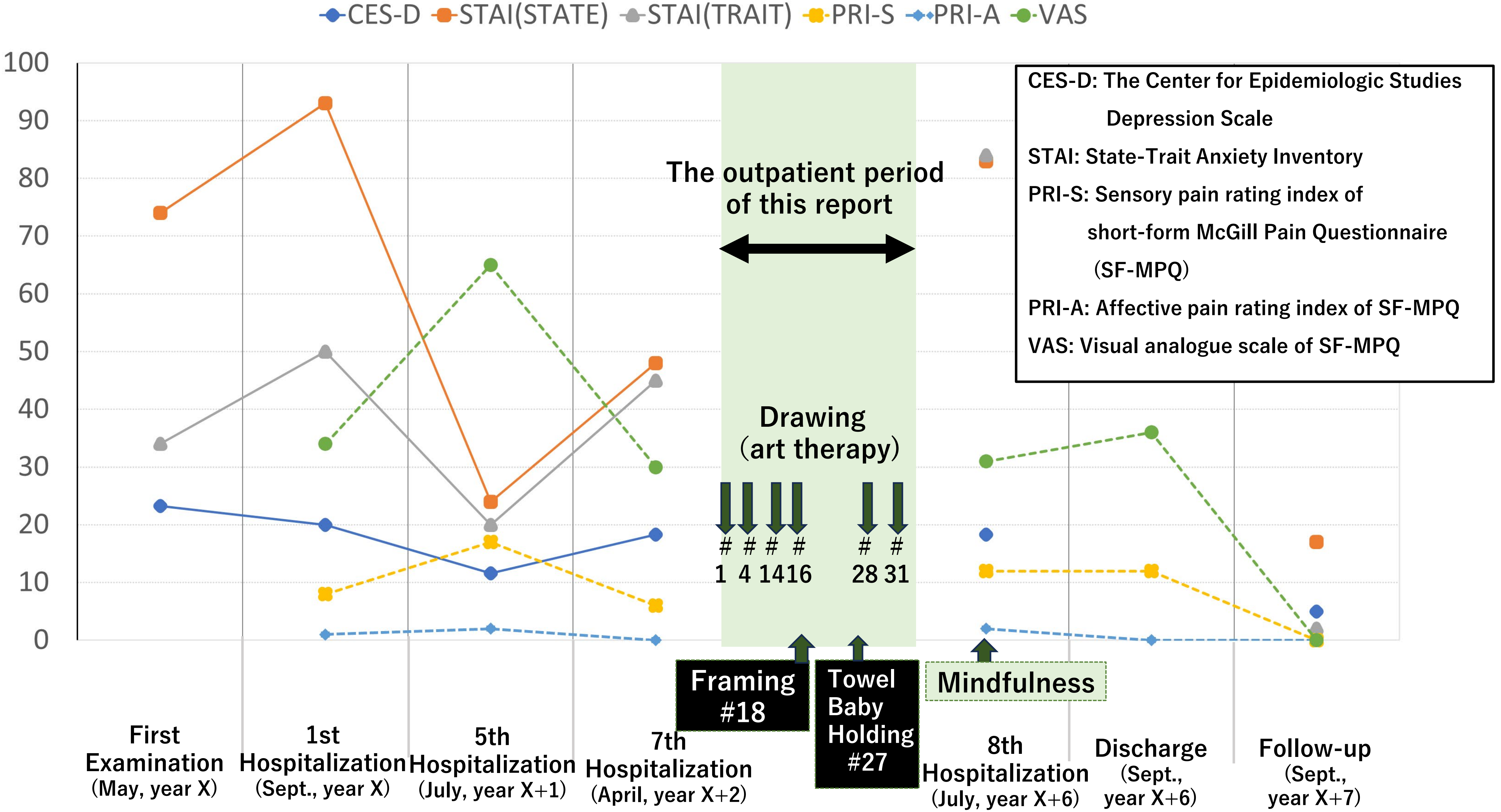

Supplement: Supplementary file 1 [file Datasheet1.pdf]
